# Promoter Polymorphism of RGS2 Gene Is Associated with Change of Blood Pressure in Subjects with Antihypertensive Treatment: The Azelnidipine and Temocapril in Hypertensive Patients with Type 2 Diabetes Study

**DOI:** 10.4061/2010/196307

**Published:** 2010-08-24

**Authors:** Ken Sugimoto, Tomohiro Katsuya, Kei Kamide, Tomomi Fujisawa, Izumi Shimaoka, Mitsuru Ohishi, Ryuichi Morishita, Toshio Ogihara, Hiromi Rakugi

**Affiliations:** ^1^Department of Geriatric Medicine and Nephrology, Osaka University Graduate School of Medicine, 2-2 B6, Yamada-oka, Suita, Osaka 565-0871, Japan; ^2^Department of Clinical Gene Therapy, Osaka University Graduate School of Medicine, Osaka 565-0871, Japan; ^3^Osaka General Medical Center, Osaka Prefectural Hospital Organization, Osaka, Japan

## Abstract

We performed a prospective study to examine the genetic effect on the response to a calcium (Ca) channel blocker, azelnidipine and an ACE inhibitor, temocapril treatment in patients with hypertension, as a part of the prior clinical trial, the Azelnidipine and Temocapril in Hypertensive Patients with Type 2 Diabetes Study (ATTEST). *Methods and Results*. All subjects who gave informed consent for genetic research were divided into two groups: the subjects treated with azelnidipine or temocapril, for 52 weeks. We selected 18 susceptible genes for hypertension and determined their genotypes using TaqMan PCR method. RNA samples were extracted from peripheral blood, and quantitative real time PCR for all genes was performed using TaqMan method. One of the polymorphisms of the RGS2 gene was extracted as being able to influence the effect of these treatments to reduce BP. At eight weeks, BP change showed a significant interaction between the A-638G polymorphism of Regulator of G protein signaling-2 (RGS2) gene and treatment with azelnidipine or temocapril. There was no gene whose expression was associated with BP phenotypes or the polymorphisms of each gene. *Conclusions*. A-638G polymorphism of the RGS-2 gene could be a predictive factor for therapeutic performance of Ca channel blockers.

## 1. Introduction

Genetic approaches may provide a powerful tool for clarifying the pathogenesis of essential hypertension. Many reports have demonstrated that gene polymorphisms of the renin-angiotensin system (RAS) are associated with hypertension. There have been some reliable reports about susceptible genes for hypertension including the results from “The Millennium Genome Project for Hypertension in Japan (2000~2005) [1, 2]; however, no convincing gene has yet been detected. Some of the genes regulating blood pressure might also be related to the response to antihypertensive medication [3, 4]. Indeed, we and other collaborators have investigated several susceptible genes related to hypertension [5–15], including genes of not only the renin-angiotensin system and sodium handling but also insulin resistance, oxidative stress, and sympathetic nervous system (described in the Section 2); however, the genes involved in the response to antihypertensive medication have not yet been identified. In addition, exhaustive gene expression analysis (transcriptome analysis) for lifestyle-related diseases has not been performed thus far. We performed a large collaboration with the study group led by Professor Katayama at Saitama Medical University to perform a randomized controlled trial called “Azelnidipine (a calcium (Ca) channel blocker) and Temocapril (an ACE inhibitor) in Hypertensive Patients with Type 2 Diabetes Study (ATTEST) [16]”, which included genetic analysis to evaluate the therapeutic effects of azelnidipine and temocapril.

The goals of this study were, first, to assess the association between polymorphisms of susceptible genes for hypertension (18 genes) and each treatment or phenotype and, second, to assess the association between expression in peripheral blood of susceptible genes for hypertension and each treatment or phenotype.

## 2. Methods

### 2.1. Study Subjects

**Figure 1:**
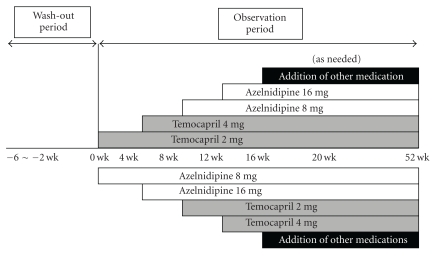
Study protocol; wk: week.

ATTEST Study, a multicenter, randomized, open-label study, was originally performed to investigate the efficacy and safety of combination therapy using the calcium channel blocker (CCB) azelnidipine and angiotensin-converting enzyme (ACE) inhibitor temocapril in hypertensive diabetics, led by Professor Katayama of Saitama Medical University. All of the subjects fulfilled the following inclusion criteria: (1) age: 30~70 years, outpatients, (2) mean systolic BP (BP): 140~180 mmHg and/or mean diastolic BP: 90~110 mmHg in washout period (four weeks) and stable BP without fluctuation in systolic BP of more than 30 mmHg or diastolic BP of more than 15 mmHg, and (3) fasting blood glucose: more than 126 mg/dl or HbA1c: more than 6.5% at 6 months before entry. These mild or moderate hypertensive subjects with diabetes were treated with the following medication: CCB: azelnidipine and ACE inhibitor: temocapril in accordance with the protocol (Figure 1). Each medication was started at a dose of 8 mg azelnidipine or 2 mg temocapril and increased to 16 mg azelnidipine or 4 mg temocapril until BP of less than 130/80 mmHg was achieved. All subjects were measured for body height, body weight, systolic BP, diastolic BP, fasting blood glucose level, triglyceride level, low density lipoprotein (LDL) cholesterol level, and serum creatinine (Cr) level.

Informed consent for genetic analysis was obtained from all subjects, and finally a total of 44 subjects were recruited in this study.

### 2.2. Selection of Susceptible Genes for Hypertension and Genotyping and Quantitative Real Time PCR (RT-PCR)

The 18 genes shown in Table 1 were selected for the current study. All of the genes were previously reported to be susceptible genes for hypertension. The genotypes of the 22 polymorphisms of the 18 susceptible genes (ACE Ins/del, ADD1 Gly460Trp (rs4961), ADIPOQ Ile164Thr, ADRB1 Ser49Gly (rs1801252) and Arg389Gly (rs1801253), ADRB2 Gly16Arg (rs1042713) and Glu27Gln (rs1042714), ADRB3 Trp64Arg (rs4994), AGT Met235Trp (rs699), AGTR1 A1166C (rs17231380), ALDH2 Glu487Lys (rs671), ARHGAP8 Arg338Gly (rs6007334), BDKRB2 T-58C, FASL G-670A, GRK4 Ala486Val (rs1801058) and Arg65Leu (rs2960306), hOGG1 Ser326Cys (rs1052133), MTHFR C677T (rs1801133), RGS2 A to G in promoter (rs3767489), RGS2 A-638G (rs2746071), SLC12A3 Arg904Gln (rs11643718), and TGFB1 Leu10Pro (rs1982073)) for which positive associations with hypertension were previously reported were successfully determined using TaqMan PCR method (Applied Biosystems Inc., Foster City, CA, USA).

According to quantitative RT-PCR analysis using cDNA extracted from peripheral blood, 11 genes were expressed in peripheral blood: ACE, ADD1, ADRB1, ADRB2, ARHGAP8, FASL, GRK4, MTHFR, RGS2, SLC12A3, and TGFB1. RNA samples were extracted from the peripheral blood of the subjects, and cDNA was made from RNA using reverse transcriptase. Quantitative real-time PCR (RT-PCR) for all the genes was performed using TaqMan PCR method to determine their expression levels.

### 2.3. Statistical Analysis

The associations between polymorphisms and clinical variables were analyzed using one-way analysis of variance (ANOVA). The difference in each genotype or allele distribution was examined by *χ* ^2^ analysis. Odd ratios were calculated as an index of the association of each genotype with the prevalence of hypertension. To assess the contribution of confounding factors, we performed multiple logistic regression analysis using the computer software application, JMP 7.0 (SAS Institute Inc., Cary, NC, U.S.A.). We focused on testing for SNP medication and gene-expression medication interactions (i.e., whether the effects of SNPs on systolic BP or diastolic BP differed between hypertensives on azelnidipine and temocapril). Multivariate analysis of variance (MANOVA) was performed to determine whether BP change showed a significant interaction of each antihypertensive medication with the genotypes of the susceptible genes.

## 3. Results

### 3.1. Baseline Characteristics of Subjects Treated with Azelnidipine and Temocapril

Baseline characteristics of the subjects treated with azelnidipine and temocapril are shown in Table 2. There was no difference in sex, age, body mass index (BMI), affected period of hypertension, systolic BP, triglyceride level, LDL cholesterol level, and serum creatinine level between the azelnidipine and temocapril groups; however, fasting glucose level was higher in the azelnidipine group than in the temocapril group at the baseline.

### 3.2. BP Reduction with Azelnidipine and Temocapril

**Figure 2:**
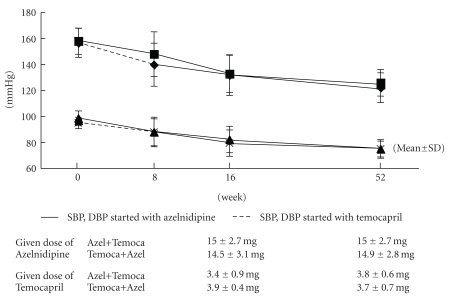
BP reduction with azelnidipine and temocapril; BP: blood pressure, SBP: systolic BP, and DBP: diastolic BP.

In the current study, the same level of BP reduction was observed in both the azelnidipine and temocapril groups, and both groups achieved a mean BP of less than 130/80 mmHg at 52 weeks. There was no difference in the dose of each treatment at 52 weeks (group started with azelnidipine: BP at 52 weeks 121.9 ± 11.4/74.7 ± 7.1 mmHg, final dose of azelnidipine 15.0 ± 2.7 mg, final dose of temocapril 3.8 ± 0.6 mg; group started with temocapril: BP at 52 weeks 125.6 ± 10.3/74.7 ± 6.1 mmHg, final dose of azelnidipine 14.9 ± 2.8 mg, final dose of temocapril 3.7 ± 0.7 mg, Figure 2).

### 3.3. Susceptible Gene Polymorphisms for Hypertension

**Figure 3:**
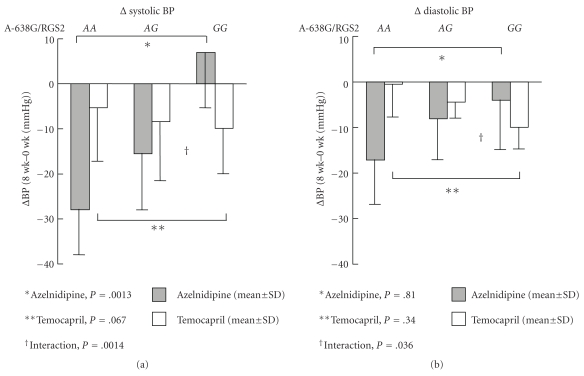
Relationship between A-638G polymorphism of RGS2 gene and BP change.

The genotypes of 22 polymorphisms of the 18 genes were successfully determined at each time point (0, 8, 16, and 52 weeks). Only the Ile164Thr polymorphism of the adiponectin gene has no mutant genotype, and the other genotype frequencies were not significantly different from the values of Hardy-Weinberg's expectation (Figure 3).

According to the analysis of the association between BP, changes of BP, and all genotypes at each time point (0, 8, 16, and 52 weeks), only the A-638G polymorphism of the regulator of G protein signaling-2 gene (*RGS2*) showed a significant association with changes in BP (ΔBP: BP at 8 weeks—BP at 0 week). As shown in Figure 3, there was a significant relationship between the A-638G polymorphism of the RGS2 gene and the changes in BP between 0 and 8 weeks in subjects with azelnidipine (Δsystolic BP: *A* *A* − 28.0 ± 10.1 mmHg, *A* *G* − 15.5 ± 12.6 mmHg, *G* *G* + 7.0 ± 12.2 mmHg, *P* = .0013; Δdiastolic BP: *A* *A* − 17.2 ± 9.8 mmHg, *A* *G* − 8.1 ± 9.0 mmHg, *G* *G* − 4.0 ± 11.0 mmHg, *P* = .067), but not in subjects with temocapril (Δsystolic BP: *A* *A* − 5.3 ± 11.8 mmHg, *A* *G* − 8.4 ± 13.0 mmHg, *G* *G* − 9.9 ± 10.0 mmHg, *P* = .81; Δdiastolic BP: *A* *A* − 0.5 ± 7.2 mmHg, *A* *G* − 4.4 ± 3.5 mmHg, *G* *G* − 10.0 ± 4.5 mmHg, *P* = .34). At 8 weeks, changes in BP showed a significant interaction between *A-638G* and treatment with azelnidipine and temocapril (Δsystolic BP, Δdiastolic BP: *P* = .0014, *P* = .036, resp., by MANOVA, after adjustment for age, sex, and BMI) (Table 3). 

**Figure 4:**
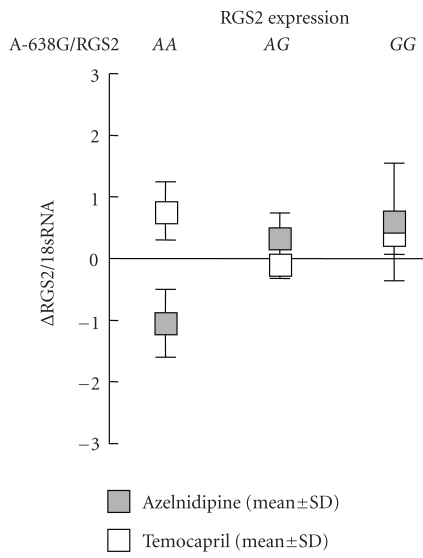
Relationship between A-638G polymorphism of RGS2 gene and RGS2 gene expression in peripheral blood.

There was no gene whose expression was associated with BP phenotypes or the polymorphisms of each gene through analysis of gene expression in peripheral blood. In terms of the RGS2 gene, the A-638G polymorphism was not related to the change of RGS2 expression between 0 and 8 weeks either in subjects with azelnidipine (ΔRGS2/18sRNA with azelnidipine: *A* *A* − 1.06 ± 2.1, *A* *G* + 0.31 ± 0.85, *G* *G* + 0.57 ± 0.44, *P* = .13, Figure 4) or in subjects with temocapril (ΔRGS2/18sRNA with temocapril: *A* *A* + 0.75 ± 1.3, *A* *G* − 0.10 ± 0.62, *G* *G* + 0.38 ± 0.44, *P* = .24, Figure 4).

## 4. Discussion

This study demonstrated the importance of pharmacogenetic research. Previously, Lynch et al. reported that the *NPPA* T2238C variant was associated with modification of antihypertensive medication effects on cardiovascular disease and BP, and TT allele carriers had more favorable outcomes when randomized to receive a CCB (amlodipine) [17]. Beitelshees et al. also demonstrated that the KCNMB1 genotype influenced responsiveness to verapamil SR and risk of adverse cardiovascular outcomes [18]. These reports could support the possibility of the existence of genes influencing drug efficacy.

Signaling by G-protein-coupled neurotransmitter receptors in the autonomic nervous system and vasoregulatory factor receptors in the periphery governs both blood pressure, by controlling the constriction and dilatation of resistance arterioles, and electrolyte and fluid balance by the kidney [19, 20]. The recently identified regulator of G-protein signaling (RGS) proteins is important in regulating signaling cascades initiated by G-protein-coupled receptors (GPCRs) activation [21]. RGS proteins facilitate the intrinsic inactivating guanosine triphosphatase reaction of G-protein a-subunits,and thereby serve as effector channel blockers. RGS2 is unique among the RGS proteins in its apparent selectivity towards Gpa, which mediates the action of mouse physiological vasoconstrictors, including norepinephrine, angiotensin II, endothelin-1, and thrombin. RGS2 can also attenuate Gi- and Gs-mediated pathways [22, 23], which can also affect blood pressure via other physiologically important agonists such as serotonin, dopamine, and bradykinin. It was recently reported that mice lacking *RGS2* exhibit a strong hypertensive phenotype (increase in SBP of 50 mmHg) and resistance vasculature [24, 25]. Both heterozygous and homozygous *RGS2*-null mice exhibited a similar level of marked hypertension, suggesting that a naturally occurring mutation that affects the level of RGS2 protein may have a significant impact on blood pressure regulation. Recently there have been two reports that genetic changes in *RGS2* are associated with a hypertensive phenotype [26, 27]. A-638G, T1026A, and 1891-1892 del TC polymorphisms were extracted on the condition the allele frequencies of these polymorphisms were >0.1, and the T1026A and 1891-1892 del TC polymorphisms of this gene were associated with hypertension in women. These findings suggest that some functional variants of the RGS2 gene might be involved in regulating blood pressure in humans. 

This ATTEST gene study revealed that one possible gene related to the effect of antihypertensive agents. In the current study, the A-638G polymorphism of the RGS2 gene was a predictive factor for therapeutic performance of a CCB, azelnidipine. Other classical candidate genes including genes associated with the renin-angiotensin system, sodium handling, vasodilatation and vasoconstriction, and the sympathetic nervous system did not show significant association between their polymorphisms and the effect of a Ca channel blocker or ACE inhibitor. In addition, there was no significant relationship between RGS2 polymorphisms and BP-related phenotypes in this study, unlike previous reports. It has not yet been reported that the promoter variant A-638G of the RGS2 gene may change RGS2 function; however, this polymorphism has the possibility to change RGS2 protein production because this polymorphism is in the promoter region. There has been no evidence supporting the idea that RGS2 could be involved in the effect of CCBs. In several previous reports, genes without a drug-metabolizing effect, such as ACE [3] or AGTR1 [4], showed a significant association between their polymorphisms and response to antihypertensive medication; however, the mechanisms have not been clarified in any of these reports. In terms of the present study, RGS2 is suggested to function as a switch to turn on or off the G protein-associated pathway, and RGS2 can regulate blood pressure through smooth muscle cells. From this viewpoint, RGS2 might have the possibility of changing the effect of CCBs, because those mainly act through SMC; however, the detailed mechanism merits further investigation in the future.

Peripheral blood mainly contains white blood cells, red blood cells, platelets, and other circulating hormones, so the distribution of genes expressed in peripheral blood has to be investigated to clarify the significance of disease susceptibility gene expression in peripheral blood. In the present study, 11 genes were expressed in peripheral blood. There was no significant relationship between gene expression including the RGS2 gene and the effect of antihypertensives and phenotypes. From this viewpoint, RGS2 expression in peripheral blood does not seem to do anything and might not be a marker for BP regulation. However, further study is needed on its clinical application as a marker of drug efficacy.

As the limitation of this study, we could not exclude the possibility of the false positive association (type I error) due to the use of small number of subjects. However, this study was carried out under the strict protocol of a clinical trial so that the reliability of the results obtained seems to be high. To confirm the effect of RGS2 gene in the tailored medicine of hypertension, further study using another panel of hypertensive subjects should be required.

In conclusion, the A-638G polymorphism of the RGS2 gene could be a predictive factor for therapeutic effectiveness of CCBs such as azelnidipine. Further research is needed to determine the optimal approach for personalizing antihypertensive medication treatment regimens according to genotype information and for achieving the best blood pressure control.

## Figures and Tables

**Table 1:** Susceptible genes for hypertension.

(1)	Angiotensin converting enzyme (ACE)
(2)	*α*-adducin (ADD1)
(3)	Adiponectin (ADIPOQ)
(4, 5, 6)	*β*1/*β*2/*β*3 adrenergic receptor (ADRB1/2/3)
(7)	Angiotensinogen (AGT)
(8)	Angiotensin II type1 receptor (AGTR1)
(9)	Aldehyde dehydrogenase 2 (ALDH2)
(10)	Rho-GTPase activating protein-8 (ARHGAP8)
(11)	Bradykinin receptor *β*2 (BDKRB2)
(12)	Fas ligand (FASL)
(13)	G protein-coupled receptor kinase 4 (GRK4)
(14)	Human 8-hydroxyguanine DNA-glycosylase (hOGG1)
(15)	Methylenetetrahydrofolate reductase (MTHFR)
(16)	Regulator of G protein signaling-2 (RGS2)
(17)	Solute carrier family 12 member 3 (SLC12A3)
(18)	Transforming growth factor-*β* (TGFB1)

**Table 2:** Baseline characteristics of subjects treated with azelnidipine and temocapril.

	Azelnidipine	Temocapril	*P* value
*n*	23	21	
Male/Female (*n*), (%)	18/5 (78/22)	15/6 (71/29)	.60
Age (years)	60.8 ± 7.9	61.0 ± 8.5	.96
BMI (Kg/m^2^)	25.6 ± 4.3	25.8 ± 3.3	.85
Period of HT (yr)	5.3 ± 5.7	9.0 ± 12.8	.21
Systolic BP (mmHg)	155.1 ± 11.1	157.2 ± 10.2	.52
Diastolic BP (mmHg)	97.8 ± 5.7	95.2 ± 5.4	.14
Fasting BG (mg/dL)	147.4 ± 27.5	132.3 ± 19.4	.04
TG (mg/dL)	127.3 ± 54.7	129.1 ± 61.3	.92
LDL-cholesterol (mg/dL)	136.1 ± 39.5	137.0 ± 39.0	.94
Serum Cr (mg/dL)	0.75 ± 0.12	0.77 ± 0.15	.58

HT: hypertension, BP: blood pressure, BG: blood glucose, TG: triglyceride, LDL: low density lipoprotein, and Cr: creatinine (mean±SD).

**Table 3:** Genotype frequencies of 22 SNPs.

SNP Name	Major Homo (*n*)		Hetero (*n*)		Minor Homo (*n*)		Hardy-Weinberg Expectation(o value)
ACE Ins/Del (I/D)	II	15	ID	23	DD	6	0.54
ADD1 Gly460Trp (G/T)	GG	10	GT	24	TT	10	0.55
ADIPOQ Ile164Thr (T/C)	TT	44	TC	0	CC	0	-
ADRB1 Ser49Gly (G/A)	GG	1	GA	6	AA	37	0.24
ADRB1 Arg389Gly (G/C)	GG	3	GC	14	CC	27	0.53
ADRB2 Gly16Arg (A/G)	AA	14	AG	18	GG	12	0.23
ADRB2 Glu27Gln (C/G)	CC	41	CG	3	GG	0	0.81
ADRB3 Trp64Arg (T/C)	TT	29	TC	13	CC	2	0.73
AGT Met235Trp (T/C)	TT	31	TC	12	CC	1	0.90
AGTR1 A1166C (A/C)	AA	36	AC	7	CC	1	0.38
ALDH2 Glu487Lys (G/A)	GG	29	GA	13	AA	2	0.73
ARHGAP8 Arg338Gly (C/G)	CC	9	CG	20	GG	15	0.63
BDKRB2 T-58C (T/C)	TT	7	TC	23	CC	14	0.63
FASL G-670A (G/A)	GG	16	GA	17	AA	11	0.15
GRK4 Ala486Val (C/T)	CC	12	CT	20	TT	12	0.55
GRK4 Arg65Leu (G/T)	GG	33	GT	9	TT	2	0.21
hOGG1 Ser326Cys (G/C)	GG	7	GC	24	CC	13	0.46
MTHFR C677T (C/T)	CC	16	CT	24	TT	4	0.24
RGS2 A to G in promoter (A/G)	AA	9	AG	22	GG	13	0.96
RGS2 A-638G (A/G)	AA	9	AG	23	GG	12	0.74
SLC12A3 Arg904Gln (G/A)	GG	39	GA	5	AA	0	0.69
TGFB1 Leu10Pro (T/C)	TT	12	TC	24	CC	8	0.51
